# First person – Mei-Fang Lin

**DOI:** 10.1242/bio.042507

**Published:** 2019-03-15

## Abstract

First Person is a series of interviews with the first authors of a selection of papers published in Biology Open, helping early-career researchers promote themselves alongside their papers. Mei-Fang Lin is first author on ‘Transcriptomic analyses highlight the likely metabolic consequences of colonization of a cnidarian host by native or non-native *Symbiodinium* species’, published in BiO. Mei-Fang conducted the research described in this article while a PhD student in David John Miller's lab at James Cook University, Australia. She is now a postdoc in the lab of Hiroshi Watanabe at the Okinawa Institute of Science and Technology, Japan, investigating cnidarian genomics and evolution.


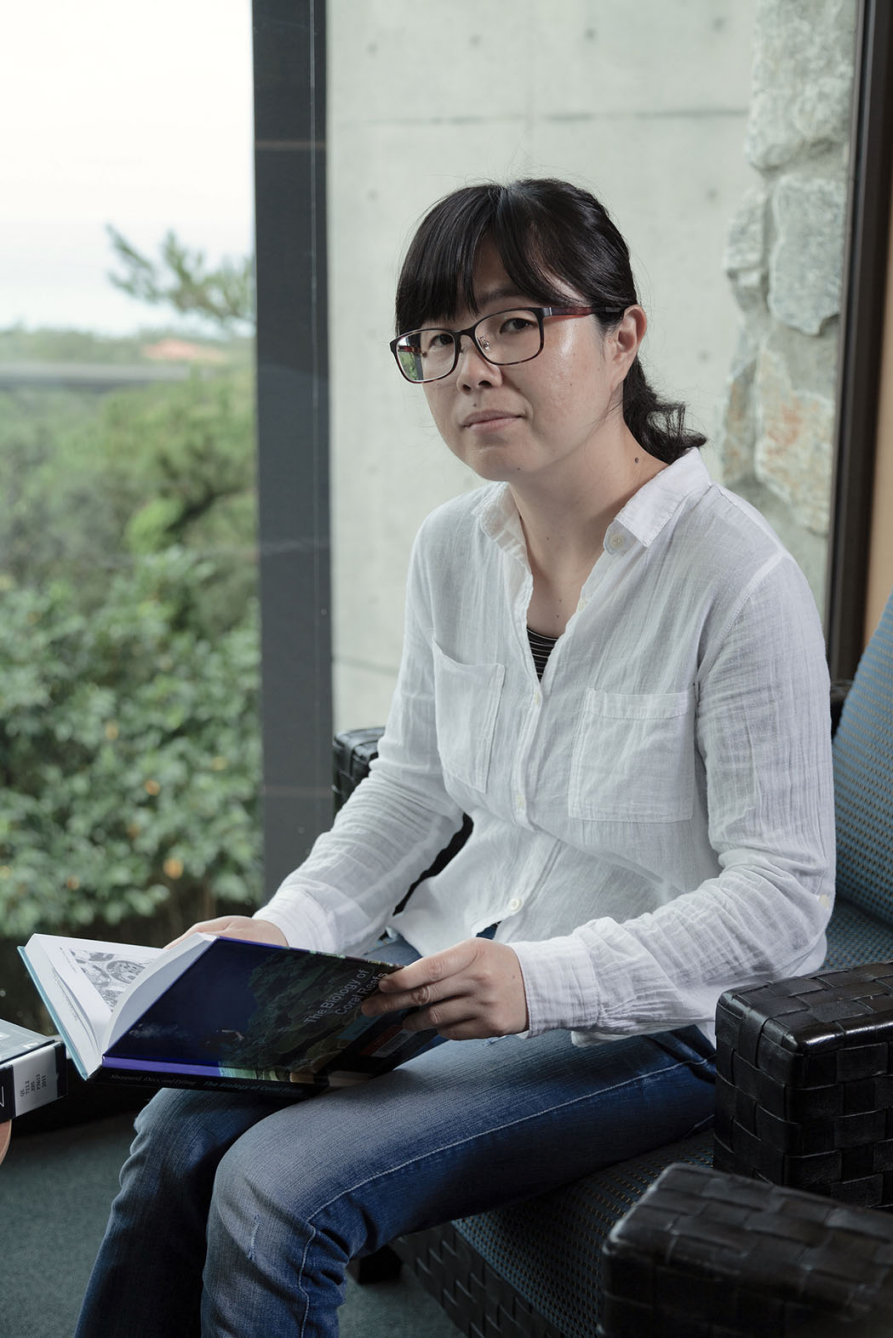


**Mei-Fang Lin**

**What is your scientific background and the general focus of your lab?**

Originally from Taiwan, I have been fascinated by the theory of evolution since I was an undergraduate student. During that time, I was introduced to systematics of red algae and the world of coral reefs. My MSc in Marine Biology at National Taiwan University was a study of coral evolution by applying molecular phylogeny. Then I joined the Biodiversity Research Centre, Academia Sinica, where I developed an interest of coral genomics. After obtaining work experience, I started my PhD journey at James Cook University, Australia, studying coral genomics and evolution under the supervision of Prof. David Miller. I am now a postdoctoral researcher at the Okinawa Institute of Science and Technology, Japan, working on evolutionary neurobiology in Prof. Hiroshi Watanabe's group. We investigate the evolution of neurons by studying early-branching animals, e.g. cnidarians, placozoas and comb jellyfish.

**How would you explain the main findings of your paper to non-scientific family and friends?**

Many marine cnidarians, e.g. corals, sea anemones and jellyfishes, are colonized by symbiotic algae of the genus *Symbiodinium.* This association plays a crucial role in their growth and health. It is believed that symbionts are expelled when the corals are under stress, but that corals are able to recover the symbionts when environmental conditions again become favorable. However, it is unclear whether different symbiont associations alter coral health. In this study, we investigated gene expression patterns of corallimorpharians, close relatives of corals, when colonized with native symbiont and non-native symbionts. We found that the different associations had different metabolic consequences, suggesting that although cnidarians are able to recover by hosting non-native symbionts, metabolic conditions are not identical to those when they harbor native symbionts.

**What are the potential implications of these results for your field of research?**

Cnidarian-*Symbiodinium* symbioses are the foundation of coral reef ecosystems. The success of symbiosis relies on optimal nutritional exchange between host and symbiont. Metabolic performance of cnidarians differs, depending upon whether they are colonized with native or non-native symbionts; thus, symbioses with different *Symbiodinium* species are not functionally equivalent. Our study has provided further knowledge of how host cnidarians change dominant *Symbiodinium* phylotypes to adapt to environmental changes and has serious implications for coral/cnidarian restoration.

“…this is the first time that cnidarians with heterologous symbiotic associations have been shown to revert back to the native *Symbiodinium* species in an artificial environment.”

**What has surprised you the most while conducting your research?**

A number of previous studies have suggested the importance of nutritional exchange for optimal performance of cnidarian-*Symbiodinium* associations. In our study, this is the first time that cnidarians with heterologous symbiotic associations have been shown to revert back to the native *Symbiodinium* species in an artificial environment. This result provided the first evidence of higher performance of a native association and host involvement in symbiont selection.

**Figure:**
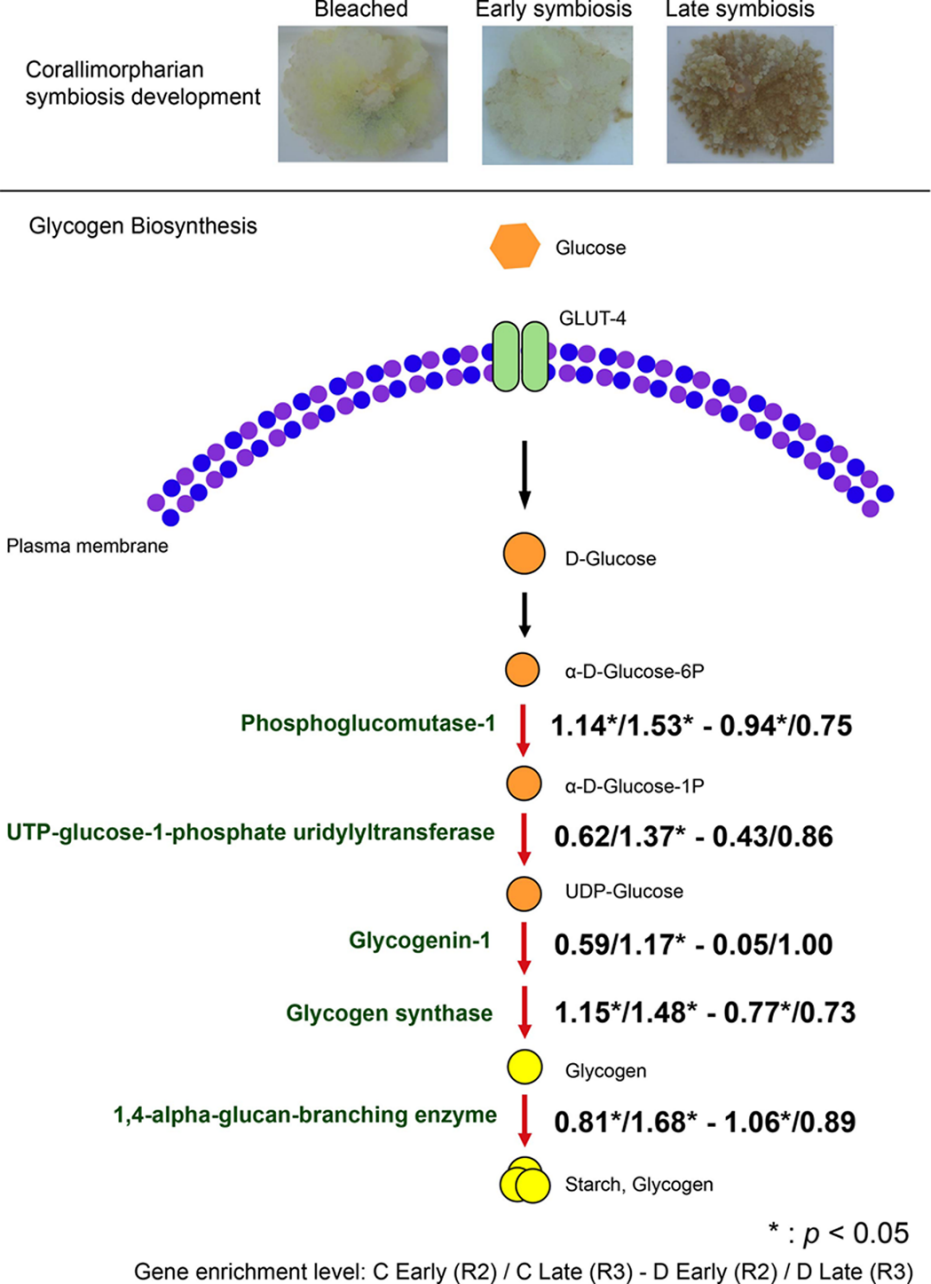
**Gene enrichment involved in the glycogen biosynthesis during symbiosis establishment.**

**What, in your opinion, are some of the greatest achievements in your field and how has this influenced your research?**

Although our understanding of cnidarian-*Symbiodinium* symbiosis remains limited, the power of high-throughput sequencing technology has inaugurated a new era in this field of study. With large-scale sequencing data, we are able to investigate processes of cnidarian-*Symbiodinium* interactions in detail. In our study, we applied transcriptomic approaches to examine transcriptional effects in the host during heterologous symbiosis. This has helped us to understand which genes are involved and regulated by the association. In the near future, the rapid development of high-throughput sequencing methods will help to reveal the complex mechanisms of cnidarian-*Symbiodinium* symbioses.

**What changes do you think could improve the professional lives of early-career scientists?**

I think diverse career advice is very helpful to early-career scientists. Many early-career scientists have received good scientific training and knowledge of their specific fields of study, but very few of them remain in academia. The lack of job security for professionals has decreased interest and dragged down professional development. Providing career advice and job search skills would assist early-career scientists in moving on and would give them more opportunities to contribute to society.

**What's next for you?**

The evolution of symbiosis is the keystone of coral diversity. Although we have spent decades investigating this process, there are still many fundamental questions unanswered. I will continue to pursue my interest in cnidarian evolution and I hope to better understand symbiotic mechanisms, and more importantly, how cnidarians cope with climate change.
